# Correction: Influenza as a molecular walker

**DOI:** 10.1039/d0sc90015j

**Published:** 2020-02-18

**Authors:** P. H. (Erik) Hamming, Nico J. Overeem, Jurriaan Huskens

**Affiliations:** Molecular Nanofabrication Group, MESA + Institute for Nanotechnology, Faculty of Science and Technology, University of Twente P.O. Box 217 7500 AE Enschede The Netherlands j.huskens@utwente.nl

## Abstract

Correction for ‘Influenza as a molecular walker’ by P. H. (Erik) Hamming *et al.*, *Chem. Sci.*, 2020, **11**, 27–36.

The authors regret that incorrect details were given for ref. 70 in the original article. The correct version of ref. 70 is given below as ref. 1.

The Royal Society of Chemistry apologises for these errors and any consequent inconvenience to authors and readers.
